# The effect of the shock index and scoring systems for predicting mortality among geriatric patients with upper gastrointestinal bleeding: a prospective cohort study

**DOI:** 10.1590/1516-3180.2021.0735.13102021

**Published:** 2022-05-09

**Authors:** Umran Dogru, Melih Yuksel, Mehmet Oguzhan Ay, Halil Kaya, Aksel Ozdemır, Yesim Isler, Mehtap Bulut

**Affiliations:** I MD. Emergency Medicine Specialist, Department of Emergency Medicine, University of Health Sciences, Bursa Yuksek Ihtisas Training and Research Hospital, Bursa, Turkey.; II MD. Associate Professor of Emergency Medicine, Department of Emergency Medicine, University of Health Sciences, Bursa Yuksek Ihtisas Training and Research Hospital, Bursa, Turkey.; III MD. Associate Professor of Emergency Medicine, Department of Emergency Medicine, University of Health Sciences, Bursa Yuksek Ihtisas Training and Research Hospital, Bursa, Turkey.; IV MD. Professor of Emergency Medicine, Department of Emergency Medicine, University of Health Sciences, Bursa Yuksek Ihtisas Training and Research Hospital, Bursa, Turkey.; V MD. Emergency Medicine Specialist, Department of Emergency Medicine, University of Health Sciences, Bursa Yuksek Ihtisas Training and Research Hospital, Bursa, Turkey.; VI MD. Emergency Medicine Specialist, Department of Emergency Medicine, University of Health Sciences, Bursa Yuksek Ihtisas Training and Research Hospital, Bursa, Turkey.; VII MD. Professor of Emergency Medicine, Department of Emergency Medicine, University of Health Sciences, Bursa Yuksek Ihtisas Training and Research Hospital, Bursa, Turkey.

**Keywords:** Emergencies, Geriatrics, Mortality, Gastrointestinal bleeding, Shock index, Rockall score, Glasgow-Blatchford score, AIMS-65 score

## Abstract

**BACKGROUND:**

Gastrointestinal (GI) bleeding is an important cause of mortality and morbidity among geriatric patients.

**OBJECTIVE:**

To investigate whether the shock index and other scoring systems are effective predictors of mortality and prognosis among geriatric patients presenting to the emergency department with complaints of upper GI bleeding.

**DESIGN AND SETTING:**

Prospective cohort study in an emergency department in Bursa, Turkey.

**METHODS:**

Patients over 65 years admitted to a single-center, tertiary emergency service between May 8, 2019, and April 30, 2020, and diagnosed with upper GI bleeding were analyzed. 30, 180 and 360-day mortality prediction performances of the shock index and the Rockall, Glasgow-Blatchford and AIMS-65 scores were evaluated.

**RESULTS:**

A total of 111 patients who met the criteria were included in the study. The shock index (P < 0.001) and AIMS-65 score (P < 0.05) of the patients who died within the 30-day period were found to be significantly different, while the shock index (P < 0.001), Rockall score (P < 0.001) and AIMS-65 score (P < 0.05) of patients who died within the 180-day and 360-day periods were statistically different. In the receiver operating characteristic (ROC) analysis for predicting 360-day mortality, the area under the curve (AUC) value was found to be 0.988 (95% confidence interval, CI, 0.971-1.000; P < 0.001).

**CONCLUSION:**

The shock index measured among geriatric patients with upper GI bleeding at admission seems to be a more effective predictor of prognosis than other scoring systems.

## INTRODUCTION

Gastrointestinal (GI) bleeding is a serious and potentially life-threatening condition that causes approximately one million hospitalizations per year in the United States alone. GI bleeding covers bleeding originating from any part of the gastrointestinal tract and may extend from the mouth to the anus.^1^ It is divided into two categories: upper and lower GI bleeding.

Upper GI bleeding is defined as bleeding in any area from the mouth to the ligament of Treitz.^2^ Patients with upper GI bleeding usually present to emergency services with hematemesis or melena, while patients who are hemodynamically unstable and have a large amount of bleeding may also present with hematochezia.^3^ Upper GI bleeding is estimated to occur in 80-150 out of every 100,000 people per year. The estimated mortality rates are between 2% and 15%.^4^ The most common risk factors are a history of upper GI bleeding, use of anticoagulants, use of high doses of non-steroidal anti-inflammatory drugs (NSAIDs) and advanced age.^2^ ^,^ ^5^

GI bleeding is the most common cause of non-traumatic hemorrhagic shock. Shock is generally accompanied by hypotension. However, not every hypotensive patient is in shock. In order to clarify the diagnosis, the “shock index”, which is higher in patients with left ventricular dysfunction and fluid loss, has been proposed. It is obtained by dividing the heart rate by the systolic blood pressure, and its normal range is considered to be 0.5-0.7. The shock index increases in cases of trauma and bleeding, in which the left ventricular stroke volume decreases.^6^ ^,^ ^7^ ^,^ ^8^ It presents great potential for determining possible short-term negative outcomes among patients with upper GI bleeding. Additionally, it may be used in emergencies to ascertain changes to the clinical picture and is as effective as other risk-scoring systems that have been suggested in the literature.^9^

Various scoring systems are used to predict prognosis and mortality among patients with upper GI bleeding. The most frequently used scoring systems for this purpose are Rockall, Glasgow-Blatchford and AIMS-65. These use clinical information and results from laboratory tests and endoscopy.^10^ The Rockall score, which has the aim of helping to discharge low-risk patients and reduce costs, was created based on criteria such as age, comorbidity, shock status, endoscopic diagnosis and findings of new bleeding, in order to predict rebleeding in patients with upper GI bleeding.^11^ The aim of the Glasgow-Blatchford scoring system is to predict the need for intervention to control bleeding. Endoscopic findings are not included in the evaluation. Scoring is between 0 and 23, and as the score increases, the need for endoscopy also increases.^12^ AIMS-65, which is an easy-to-remember and simple scoring system, provides a risk score for predicting in-hospital mortality, length of stay and cost, for patients with acute upper GI bleeding. It is based on the patient’s age, systolic blood pressure, mental status and laboratory data.^13^

## OBJECTIVE

The aim of this study was to investigate whether the shock index and other scoring systems measured at admission to the emergency department are effective predictors of mortality and prognosis among geriatric patients with upper GI bleeding.

## METHODS

### Patient selection and location

This study was carried out in the Department of Emergency Medicine, University of Health Sciences Turkey, Bursa Yuksek Ihtisas Training and Research Hospital, with approval from the clinical research ethics committee of the same hospital (protocol number: 2011-KAEK-25-2019/05-02; date: May 8, 2019).

In this study, patients over 65 years of age with a diagnosis of upper GI bleeding who presented to the Department of Emergency Medicine, University of Health Sciences Turkey, Bursa Yuksek Ihtisas Training and Research Hospital, between May 8, 2019, and April 30, 2020, were prospectively examined.

### Exclusion criteria

- Patients aged under 65 years
- Patients with lower GI bleeding
- Upper GI bleeding due to traumatic causes
- Patients who did not undergo endoscopy
- Patients in whom upper GI bleeding was not detected, according to the results from endoscopy

### Inclusion criteria

- Patients aged 65 and over
- Patients with upper GI bleeding, according to the results from endoscopy

### Methods and measurements

A total of 128 patients were included in the study. Two of the patients were excluded because endoscopy could not be performed; five were excluded because no focus of bleeding could be detected through endoscopy; and ten patients were excluded because they could not be reached. Thus, a total of 111 patients over the age of 65 years who met the criteria and were diagnosed with upper GI bleeding through the tests and examinations were included in the study.

The patients’ vital signs and laboratory findings, and especially their demographic information, pulse rate and systolic and diastolic blood pressures, were recorded at admission. The “shock index” was calculated by dividing the patients’ pulse rate at the time of first admission by the systolic blood pressure. The patients’ existing comorbidities, current medications and endoscopy results were followed up and recorded on the case report forms.

All patients underwent endoscopy at the University of Health Sciences Turkey, Bursa Yuksek Ihtisas Training and Research Hospital. Upper GI bleeding due to peptic ulcer was recorded according to the forest classification. The Rockall, Glasgow-Blatchford and AIMS-65 scores were calculated for all patients.

The study endpoints were defined as mortality within 30 days, within 180 days and within 360 days. Consent and contact information were obtained from the patients or their relatives. The patient and/or patient’s relatives were called on days 30, 180 and 360 to get information about his or her latest status, following the outcome from the emergency department.

### Statistical analysis

IBM SPSS Statistics for Windows, version 21.0, released 2012 (IBM Corp., Armonk, New York, United States), was used for statistical analysis. Descriptive statistics were expressed as the mean ± standard deviation or the median plus interquartile range (IQR) (25%-75%), while categorical variables were expressed as the number and percentage (%). The Kolmogorov-Smirnov test was used to test the normality of the distribution of the data. The assumption of homogeneity of variances was investigated using Levene’s test.

The significance of differences between the groups, in terms of continuous numerical variables in which the statistical assumptions of parametric tests were met, was evaluated using Student’s t test. The significance of differences, regarding continuous numerical variables in which the statistical assumptions of parametric tests were not met, was investigated using the Mann-Whitney U test. Spearman’s correlation analysis was used to evaluate the relationship between variables with nonparametric distribution.

A receiver operating characteristic (ROC) curve was drawn to investigate the 30, 180 and 360-day mortality prediction performances of the shock index and Rockall, Glasgow-Blatchford and AIMS-65 scores. Logistic regression analysis was performed to determine the factors affecting mortality. The results were reported with the 95% confidence interval (CI), and P < 0.05 was considered statistically significant.

## RESULTS

A total of 111 patients were included in the study. The patients’ median age was 76 years (IQR 25-75: 69-82), and 72 (64.9%) of them were male. Among all the patients, 97 (87.4%) had a history of drug use. The most commonly used drug was acetylsalicylic acid (ASA), which was used by 33.3%. There were 100 patients (90.1%) with a history of comorbidities. The most common comorbidities were hypertension (HT) (42.3%) and coronary artery disease (CAD) (38.7%), respectively. Seventeen of the patients (15.3%) died within 30 days while the 360-day mortality rate was 38.7% (Table 1). The median heart rate of the patients was 97/min (IQR 25-75: 83-118), the median systolic blood pressure (SBP) value was 108 mmHg (IQR 25-75: 90-126), and the mean shock index was 0.996 ± 0.389 (Table 2).

**Table 1.** Clinical and demographic data

Variables		n	%
Gender	Female	39	35.1
	Male	72	64.9
Hematemesis	No	63	56.8
	Yes	48	43.2
Melena	No	14	12.6
	Yes	97	87.4
History of drug use	No	14	12.6
	Yes	97	87.4
LMWH	No	106	95.5
	Yes	5	4.5
Clopidogrel	No	96	86.5
	Yes	15	13.5
Factor Xa inhibitor	No	99	89.2
	Yes	12	10.8
Warfarin	No	96	86.5
	Yes	15	13.5
ASA	No	74	66.7
	Yes	37	33.3
NSAIDs	No	95	85.6
	Yes	16	14.4
PPI/H2-receptor antagonists	No	81	73
	Yes	30	27
Comorbidities	No	11	9.9
	Yes	100	90.1
DM	No	83	74.8
	Yes	28	25.2
HT	No	64	57.7
	Yes	47	42.3
AF	No	99	89.2
	Yes	12	10.8
CAD	No	68	61.3
	Yes	43	38.7
CHF	No	98	88.3
	Yes	13	11.7
CVDs	No	105	94.6
	Yes	6	5.4
COPD/Asthma	No	100	90.1
	Yes	11	9.9
Liver cirrhosis	No	105	94.6
	Yes	6	5.4
Malignancy	No	101	91
	Yes	10	9
Other disorders	No	74	66.7
	Yes	37	33.3
Outcome from emergency	Admission	96	86.5
	Discharge	11	9.9
	Referral	4	3.6
30-day mortality	No	94	84.7
	Yes	17	15.3
180-day mortality	No	77	69.4
	Yes	34	30.6
360-day mortality	No	68	61.3
	Yes	43	38.7
	Total	111	100

LMWH = low molecular weight heparin; ASA = acetyl salicylic acid; NSAIDs = nonsteroid anti-inflammatory drugs; PPI = proton pump inhibitor; DM = diabetes mellitus; HT = hypertension; AF = atrial fibrillation; CAD = coronary artery disease; CHF = congestive heart disease; CVDs = cerebrovascular diseases; COPD = chronic obstructive pulmonary disease.

**Table 2.** Frequency table of variables

Variables	Value
GCS, median (IQR 25-75)	15 (15-15)
SBP, median (IQR 25-75)	108 (90-126)
DBP, median (IQR 25-75)	70 (60-78)
Fever, mean ± SD	36.53 ± 0.64
SpO_2_, median (IQR 25-75)	96 (95-98)
Pulse, median (IQR 25-75)	97 (83-118)
Shock index, mean ± SD	0.996 ± 0.389
Rockall score, median (IQR 25-75)	5 (4-5)
Glasgow-Blatchford score, median (IQR 25-75)	11 (8-12)
AIMS-65 score, median (IQR 25-75)	2 (1-2)

GCS = Glasgow coma scale; SBP = systolic blood pressure; DBP = diastolic blood pressure; IQR = interquartile range; SD = standard deviation; SpO_2_ = oxygen saturation.

The Mann-Whitney U test was performed to investigate whether there were any differences in the patients’ median shock index or Rockall, Glasgow-Blatchford and AIMS-65 scores, with regard to 30, 180 and 360-day mortality. The results showed that the shock index and AIMS-65 score were significantly different among patients who died within 30 days (P < 0.001 and P < 0.05). Additionally, the shock index, Rockall score and AIMS-65 score were found to be significantly different in patients with 180-day mortality (P < 0.001, P < 0.001 and P < 0.05). Lastly, the shock index, Rockall score and AIMS-65 score were found to be significantly different among patients with 360-day mortality (P < 0.001, P = 0.001 and P < 0.05) (Table 3).

**Table 3.** Mortality analysis on variables using Mann-Whitney U test

	30-day mortality	n	Median (IQR 25-75)	P value	180-day mortality	n	Median (IQR 25-75)	P value	360-day mortality	n	Median (IQR 25-75)	P-value
Shock index	No	94	0.76 (0.67-1.18)	< 0.001	No	77	0.73 (0.64-0.91)	< 0.001	No	68	0.70 (0.63-0.83)	< 0.001
	Yes	17	1.53 (1.26-1.76)		Yes	34	1.43 (1.24-1.60)		Yes	43	1.36 (1.22-1.56)	
	Total	111	0.86 (0.68-1.28)		Total	111	0.86 (0.68-1.28)		Total	111	0.86 (0.68-1.28)	
Rockall score	No	94	4.00 (4.00-5.00)	> 0.05	No	77	4.00 (3.50-5.00)	< 0.001	No	68	4.00 (3.00-5.00)	< 0.05
	Yes	17	5.00 (5.00-5.50)		Yes	34	5.00 (5.00-6.00)		Yes	43	5.00 (5.00-6.00)	
	Total	111	5.00 (4.00-5.00)		Total	111	5.00 (4.00-5.00)		Total	111	5.00 (4.00-5.00)	
Glasgow-Blatchford score	No	94	11.00 (8.00-12.00)	> 0.05	No	77	11.00 (8.00-11.00)	> 0.05	No	68	11.00 (8.00-11.75)	> 0.05
	Yes	17	11.00 (8.50-12.50)		Yes	34	11.00 (8.75-13.00)		Yes	43	11.00 (8.00-12.00)	
	Total	111	11.00 (8.00-12.00)		Total	111	11.00 (8.00-12.00)		Total	111	11.00 (8.00-12.00)	
AIMS-65 score	No	94	1.50 (1.00-2.00)	< 0.05	No	77	1.00 (1.00-2.00)	< 0.05	No	68	1.00 (1.00-2.00)	< 0.05
	Yes	17	2.00 (1.50-3.00)		Yes	34	2.00 (1.00-2.25)		Yes	43	2.00 (1.00-2.00)	
	Total	111	2.00 (1.00-2.00)		Total	111	2.00 (1.00-2.00)		Total	111	2.00 (1.00-2.00)	

IQR = interquartile range.

**Figure 1.**
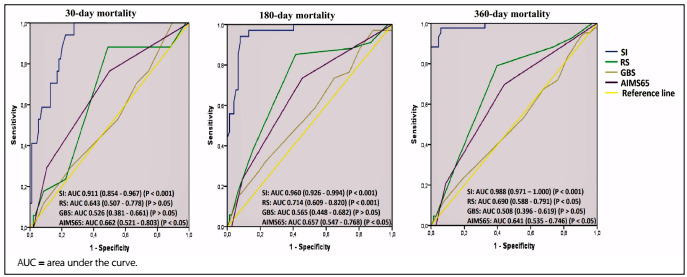
Receiver-operating characteristic curves of the shock index (SI), Rockall score (RS), Glasgow-Blatchford score (GBS) and the AIMS-65 scores for predicting 30, 180 and 360-day mortality.

The diagnostic value of the patients’ shock index and Rockall, Glasgow-Blatchford and AIMS-65 scores for 30, 180 and 360-day mortality were analyzed using ROC. For 30-day mortality, the area under the curve (AUC) for the shock index was 0.911 (P < 0.001) while the AUC for the AIMS-65 score was 0.662 (P < 0.05). For 180-day mortality, the AUC for the shock index was found to be 0.960 (P < 0.001), the AUC for the Rockall score was 0.714 (P < 0.001) and the AUC for the AIMS-65 score was 0.657 (P < 0.05). For 360-day mortality, the AUC for the shock index was 0.988 (P < 0.001), the AUC for the Rockall score was 0.690 (P < 0.05) and the AUC for the AIMS-65 score was 0.641 (P < 0.05) (Figure 1).

When the cutoff value of the shock index for 30-day mortality was 1.240, the sensitivity was found to be 82.4% and the specificity was 81.9%. When the cutoff value of the AIMS-65 score was 1.5, the sensitivity was found to be 76.5% and the specificity was 50.0%. When the cutoff value of the shock index for 180-day mortality was 1.205, the sensitivity was 91.2% and the specificity was 92.2%. When the cutoff value of the Rockall score was 5.5, the sensitivity was found to be 38.2% and specificity was 61.8%. When the cutoff value of the shock index for 360-day mortality was 1.06, the sensitivity was 95.3% and the specificity was 94.1%. Accordingly, it can be seen that the performance of the shock index was significantly better than that of the other scoring systems (Table 4).

**Table 4.** Cutoff values for the shock index, Rockall score, Glasgow-Blatchford score and AIMS-65 score for predicting 30, 180 and 360-day mortality

Variables	AUC (95% Cl)	P	Risk factor	Cutoff value	Sensitivity %	Specificity %
30-day mortality	0.911 (0.854-0.967)	< 0.001	Shock index	1.125	94.1	72.3
				1.240	82.4	81.9
				1.265	76.5	83.0
	0.643 (0.507-0.778)	> 0.05	Rockall score	3.5	88.2	22.3
				4.5	88.2	51.1
				5.5	23.5	77.7
	0.526 (0.381-0.671)	> 0.05	Glasgow-Blatchford score	8.5	76.5	25.5
				9.5	70.6	33.0
				10.5	52.9	44.7
	0.662 (0.521-0.803)	< 0.05	AIMS-65 score	1.5	76.5	50.0
				2.5	29.4	89.4
180-day mortality	0.960 (0.926-0.994)	< 0.001	Shock index	1.100	97.1	87.0
				1.170	82.4	81.9
				1.205	91.2	92.2
	0.714 (0.609-0.820)	< 0.001	Rockall score	3.5	88.2	11.8
				4.5	85.3	14.7
				5.5	38.2	61.8
	0.565 (0.448-0.682)	> 0.05	Glasgow-Blatchford score	8.5	76.5	23.5
				9.5	73.5	26.5
				10.5	58.8	41.2
	0.657 (0.547-0.768)	< 0.05	AIMS-65 score	1.5	73.5	26.5
				2.5	23.5	76.5
360-day mortality	0.988 (0.971-1.000)	< 0.001	Shock index	0.955	97.7	91.2
				1.060	95.3	94.1
				1.125	90.7	95.6
	0.690 (0.588-0.791)	< 0.05	Rockall score	3.5	88.4	26.5
				4.5	79.1	60.3
				5.5	32.6	83.8
	0.508 (0.396-0.619)	> 0.05	Glasgow-Blatchford score	8.5	72.1	23.5
				9.5	67.4	32.4
				10.5	53.5	44.1
	0.641 (0.535-0.746)	< 0.05	AIMS-65 score	1.5	69.8	55.9
				2.5	20.9	91.2

AUC = area under the curve; CI = confidence interval.

Logistic regression analysis was performed using variables of gender, comorbidities and drug use history, which were thought to have an effect on 360-day mortality. The history of drug use was found to be an effective factor for diagnosing 360-day mortality (Exp beta = 6.489; 95% CI, 1.607-26.208; P = 0.009) (Table 5).

**Table 5.** Analysis of variables using logistic regression

Variables	B	S.E.	Wald	df	P	Exp(B)	95% CI	
							Lower	Upper
Gender	-278	438	402	1	526	757	321	1.789
Drug use	1.870	712	6.895	1	009	6.489	1.607	26.208
Comorbidities	-1.747	926	3.559	1	059	174	028	1.070
Constant	-458	274	2.796	1	094	632		

B = coefficient; S.E. = standard error; df = degrees of freedom; Exp(B) = exponentiation of the B coefficient; CI = confidence interval.

## DISCUSSION

The history of drug use has an important place in the etiology of patients with upper GI bleeding. ASA and NSAIDs cause bleeding by inhibiting platelet aggregation and causing damage to the GI mucosa.

In a study by Loperfido et al., aspirin use was shown to be in first place among the causes, in patients presenting with upper GI bleeding.^14^ In a study by Laursen et al., use of ASA took first place with a rate of 41%.^15^ In our study, 87.4% of the patients had a history of drug use. The most commonly used drug was ASA, by 33.3% of the patients. Another important finding from our study was that the drugs used by the patients were an independent factor for 360-day mortality (odds ratio, OR = 6.489; 95% CI, 1.607-26.208; P = 0.009). Additionally, the most commonly used drug among the patients was ASA, which was consistent with the findings in the literature.

One of the most important factors that increase mortality and morbidity in cases of upper GI bleeding is the patients’ existing comorbidities. In a prospective study conducted by Palmer, involving 14,000 people, the most common comorbidities were found to be HT and CAD.^16^ In a prospective study by Köksal et al., in which patients with upper GI bleeding were examined, the most common comorbidity was found to be chronic liver disease, with a rate of 30%.^17^ Stanley et al., on the other hand, showed that CAD was the most common comorbidity in patients with upper GI bleeding.^18^ In our study, 90.1% of the patients had a history of comorbidities. The most common comorbidities were HT (42.3%) and CAD (38.7%), respectively. Examination of the literature shows that there are differences in terms of comorbidities. The reason for this may be the distribution of the frequency of the disease according to geographical region and the differences in the age groups of the patients.

The shock index is obtained by dividing the heart rate by the systolic blood pressure, and its normal value is between 0.5 and 0.7. When the shock index is greater than 0.9, presence of conditions that cause a decrease in left ventricular stroke volume, such as sepsis, trauma or bleeding, needs to be considered.^6^ ^,^ ^7^ ^,^ ^8^ In patients with upper GI bleeding, a decrease in left ventricular volume due to the amount of bleeding causes an increase in the shock index. In a study conducted by Jung et al. in 2019, the mean shock index was found to be 0.72 in patients with upper GI bleeding.^10^ Rassameehiran et al. observed that mortality and the need for transfusion became greater among patients with upper GI bleeding when the shock index was above 0.78. They also claimed that the shock index would be a good predictor for determining the short-term negative outcomes of patients with upper GI bleeding.^9^ In our study, the mean shock index was found to be 0.996 ± 0.389. We believe that the value that we found differed from what had been reported in the literature because the population examined was 65 years of age and over, comorbidities that cause mortality occurred more frequently in these patients and the drugs used by the patients may have had an effect.

Considering mortality, which was the most important endpoint of our study, we observed that the results in the literature differed between studies. The mean mortality among patients with upper GI bleeding was reported to be 2%-15% in the literature.^4^ Robertson et al. found that the in-hospital mortality rate due to upper GI bleeding was 4.2%, while Budimir et al. indicated that the 30-day mortality rate due to peptic ulcer bleeding was 5.2%.^19^ ^,^ ^20^ Additionally, Yaka et al. found that the in-hospital mortality rate due to upper GI bleeding was 7.1%.^21^ Similarly, in a study by Stanley et al., the 30-day mortality rate was found to be 7%.^18^ Most of the studies in the literature investigated in-hospital or 30-day mortality. In our study, in addition to 30-day mortality, we also examined 180 and 360-day mortality rates. We think that these results will contribute to the literature. In addition, the mortality rates in our study were generally higher, contrary to the data in the literature. We think that this was because the population that we examined consisted of elderly patients and because they had high numbers of comorbidities.

Various scoring systems are used to predict prognosis and mortality among patients with upper GI bleeding. The most commonly used ones are the Rockall, Glasgow-Blatchford and AIMS-65 scores. These are obtained using the clinical information, laboratory data and endoscopy results of the patients.^10^ ^,^ ^19^ In the study by Robertson et al., the AIMS-65 score was found to be better than the Glasgow-Blatchford and Rockall scoring systems for predicting mortality and the need for intensive care.^19^ In the study by Laursen et al., it was shown that the Glasgow-Blatchford and Rockall scoring systems were not useful for predicting 30-day mortality.^15^ A study by Bryant et al. on 708 patients showed that the Glasgow-Blatchford score was better than the Rockall score for predicting re-bleeding, need for surgery, need for transfusion and mortality.^22^ Wang et al. considered that all three scoring systems were inadequate.^23^ In a study on 3,012 patients, Stanley et al. found that the Glasgow-Blatchford score was better than the Rockall and AIMS-65 scores for predicting intervention or mortality.^18^ In the study by Jung et al. in 2019, comparing the shock index and the Rockall, Glasgow-Blatchford and AIMS-65 scores, they found that the Glasgow-Blatchford score and shock index were better for predicting possible adverse events.^10^ In a study by Tang et al., the AIMS-65 and Glasgow-Blatchford scores were clinically more useful for predicting 30-day mortality than the pre-endoscopic Rockall and Baylor scores, among patients with acute upper GI bleeding presenting to emergency services.^24^ In a review of 16 studies. Ramaekers et al. claimed that these scoring systems were not strong and that their use was not recommended in clinical practice.^25^

There is not enough data in the literature for the shock index, with regard to predicting mortality among geriatric patients with upper GI bleeding. In a retrospective study by Rassameehiran et al., the shock index was compared with other scoring systems and was found to be the best predictor of the need for endoscopic treatment among patients with acute upper GI bleeding.^9^ In a study by Saffouri et al., the shock index was found to have weaker performance than other scoring systems for predicting 30-day mortality among patients with acute upper GI bleeding.^26^

In our study, we found that the shock index and AIMS-65 score had statistically better results than the Rockall and Glasgow-Blatchford scores for predicting 30-day mortality. Similarly, we found that the shock index and AIMS-65 and Rockall scores had statistically better results than the Glasgow-Blatchford score for predicting the 180-day and 360-day mortality rates. Both the shock index and the AIMS-65 score outperformed other scoring systems for predicting mortality within 30, 180 and 360 days.

However, we believe that the fact that the patients included in our study were older than 65 years may have affected the success of the AIMS-65 score. As seen in the literature, there is no consensus on the effectiveness of the shock index and other scoring systems for predicting mortality and other possible complications among patients with upper GI bleeding. Saffouri et al. found that the performance of the shock index was weak with regard to predicting the 30-day mortality rate in their study.^26^ We think that the fact that the patients included in their study were younger and had less comorbidity may have caused that result. We also believe that the shock index could not be evaluated as a significant predictor of mortality in the study by Saffouri et al. due to the hemodynamical instability of their patients at the time of admission, relatively young age of the patients and presence of cardiovascular compensation.

In our study, we found that the shock index was much more effective for predicting 30, 180 and 360-day mortality, which were the endpoints of the study, than the other scoring systems. In particular, both the AUC and the cutoff values of the shock index were statistically more significant than other scoring systems. When the cutoff value for the shock index regarding 30-day mortality was 1.240, the sensitivity was found to be 82.4% and the specificity was 81.9%. When the 180-day mortality cutoff value was 1.205, the sensitivity was found to be 91.2% and the specificity was 92.2%. Lastly, when the 360-day mortality cutoff value was 1.06, the sensitivity was found to be 95.3% and the specificity was 94.1%. We believe that these cutoff values determined for the shock index will have a major role in the treatment and follow-up of geriatric patients with upper GI bleeding.

### Limitations

The most important limitation of our study was that it was a single-center study. In addition, we think that the relatively small number of patients was another important limitation. In order to obtain results with greater accuracy and reliability, further multicenter studies with larger populations would be required.

## CONCLUSION

The shock index, which is a simple, inexpensive and noninvasive parameter that can be obtained only from vital signs, is a more effective predictor for prognosis than other scoring systems, among geriatric patients with upper GI bleeding presenting to emergency departments.
